# Indian election data for polling stations and villages: National elections 2009–2019

**DOI:** 10.1038/s41597-025-05418-6

**Published:** 2025-07-01

**Authors:** Francesca R. Jensenius, Pradeep Chhibber, Sanjeer Alam, Pranav Gupta, Madhavan Somanathan

**Affiliations:** 1 Department of Political Science, University of Oslo, Oslo, Norway; 2 Department of Political Science, University of California, Berkeley, USA; 3 Centre for the Study of Developing Societies, Delhi, India

**Keywords:** Politics, Government

## Abstract

This paper describes the creation of a dataset covering polling-station-level election returns from the 2009, 2014, and 2019 Indian general elections. Indian elections are the largest in the world, and parliamentary constituencies are enormous. Within-constituency analysis is therefore crucial to address important political questions. Polling stations are the lowest level at which election returns are released. We have collected, cleaned, and standardized data on votes cast for each candidate across about 500,000 polling stations in 11 states for 2009 and 2014 and about 162,000 polling stations from one state for 2019. Polling-station numbers change between elections, while census units are more stable and can be traced over time. Using census indicators, we manually link polling stations to villages and towns in their catchment area, making it possible to merge the election data with census data and to observe local-level voting patterns over time. Our manual approach enabled us to link about 95% of the polling stations to corresponding census units and ensured a high degree of data reliability.

## Background & Summary

Indian elections are the largest in the world. For elections to the lower house of the parliament (the *Lok Sabha*), votes are cast for competing candidates in 543 single-member territorial districts (constituencies) across India’s states and union territories. In the 2024 national elections, more than 968 million people were eligible to vote, and votes were cast in more than one million polling stations.

Not long ago, studies of Indian elections generally examined election returns at the national, state, or constituency levels for single states or elections. Recent efforts to collate and standardize constituency-level data have made it possible to look at electoral patterns across constituencies and states and over time^1,2^. But parliamentary constituencies (PCs) in India are enormous geographically and demographically and incredibly diverse. After the most recent delimitation in 2008, PCs had an average population of about 2 million, with about 1.3 million eligible voters^2,3^. By 2024, this figure had grown to a population of about 2.7 million (1.8 million eligible voters). In other words, each Indian PC is larger than many decently sized countries.

This paper describes the creation of a dataset that allows for within-constituency analysis of voting patterns at the local level. The election returns we have collated are for polling stations (PSs) – the lowest level of aggregation for which election returns are available. Since polling-station identifiers change over time and this is a unit of observation for which no other data are collected, we linked polling stations to the smallest units in the 2011 census: the village and census town. Linking polling stations to the 2011 census units makes it possible to observe voting patterns for the same units over time and merge local-level election data with census data and all other data that include census indicators or can be geo-linked to census units.

Although our original ambition was to assemble polling-station-level voting data linked to census indicators for all of India, this ambition had to be scaled back owing to limitations in data availability and challenges related to linking location names across various data sources. The dataset we introduce here still represents the most comprehensive effort of its kind to date: it includes election returns for about 500,000 polling stations in 2009 and 2014. These were about 95% of the polling stations in 11 large Indian states, amounting to around 60% of India’s polling stations in these elections. We also cover 162,000 (99.5%) of the polling stations in India’s largest state, Uttar Pradesh, in 2019, encompassing about 16% of India’s polling stations.

Our data can be used at the polling-station level or collapsed to the level of census units: we identified the corresponding villages or towns for 95% of the polling stations we had data on in 2009 and 98% in 2014. On average, there are about 1.4 PSs per census unit. Collapsing the election data to the census-unit level allows us to estimate voting patterns in more than 330,000 villages and towns in 2009 and about 360,000 villages and towns in 2014. For 2019, we were able to identify the village or town of almost 140,000 PSs, which amounts to more than 83,000 villages and towns when collapsed to the census-unit level.

Within-constituency voting data are crucial to answer several important questions about Indian elections. As noted above, the enormous size of Indian PCs makes it challenging to conclude much about local levels of competitiveness and party support since there is a serious risk of ecological fallacies when observing data at such a high level of aggregation. Social divisions in India are also extremely local^4–6^. Even today, political parties build their support from the village upwards. They stress door-to-door campaigning and, to the extent possible, appoint party workers for each polling station. When combined with census data, polling-station-level election returns can be used to assess the links between salient social divisions such as caste, class, and religion, and it is also possible to look at associations between voting patterns and the provision of public and private goods since a broad range of development indicators are either recorded in the census itself or in data that can be linked to census indicators.

Whereas there have been numerous efforts to create precinct-level data on elections in the USA^7–10^, these datasets cover only some election cycles, and there is still a paucity of such data from other parts of the world: many countries do not release election data at a granular level, and even where countries do release such data, the data are generally cumbersome to work with. We are aware of only a handful of studies working with within-constituency election data in Europe^11–13^, and none from other parts of the world. Given how large India is, we believe our dataset is not only one of the few datasets on polling-station-level election returns anywhere in the world but also the largest in terms of the number of polling stations covered. The closest in size to ours is that of Baltz *et al*.^10^, who cover about 170,000 precincts across three elections. They do, however, include election returns for candidates competing for various public offices, making theirs a comprehensive and impressive dataset.

## Methods

Polling stations are where votes are cast in Indian elections. According to the Handbook For Returning Officers (the person in charge of conducting the election according to the Representation of the People Act 1951) published by the Election Commission of India (ECI) in 2014^14^, there should be approximately one PS for every 1,000 eligible voters. The PSs are located so that voters do not have to walk more than 2 km to reach them, and they are often set up in government schools or other government buildings. Therefore, the number of electors registered to a PS depends on the sizes of villages and towns and the distances between them. As of 2014, the guideline was that between 300 and 1,200 people could vote in one PS in rural areas (300–1,400 in urban areas). Exceptions to these rules are made in sparsely populated and hilly areas^15^, and there are often newspaper stories around election time about the ECI transporting voting machines on the backs of donkeys out to particularly remote areas to allow a handful of voters there to cast their votes. If the population grows across the limit of stipulated voters by the time of the elections, a PS is split into a main PS (indicated with a serial number) and an auxiliary PS (indicated by a letter added to the main serial number). The main and auxiliary PSs are located in the same place as far as possible. Generally, up to two PSs can be located in the same building in rural areas (four in urban areas).

**Fig. 1:**
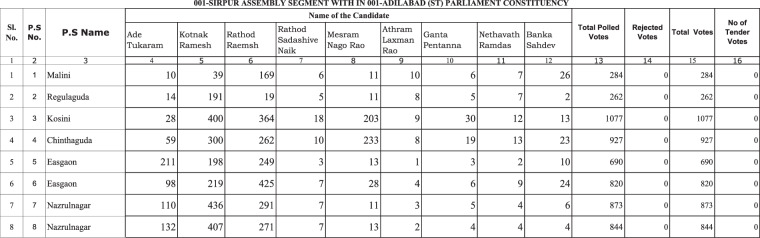
Excerpt from the Form-20 data for Andhra Pradesh from the 2009 elections.

The ECI releases PS-level election returns in a table format called Form-20 data, one table for each state assembly constituency – smaller constituencies nested within parliamentary constituencies from which candidates run in state-level elections. These tables are made public through the state-specific websites of the ECI as searchable or non-searchable PDFs and sometimes in Excel format. The tables include the serial number of the state assembly constituency in which polling stations are located, the serial number of the polling station, the total number of voters in the polling stations, and the votes cast for each candidate running for election in that particular parliamentary constituency (about 16 candidates on average). Figure 1 provides an example of how the Form-20 data appear in PDF format.

The Form-20 data do not include information about the location of the PSs, or the catchment area that they cover for eligible voters. This information can be accessed through the Electoral Rolls (ERs) – an overview of registered voters in each PS released online in PDF format before every election. The ERs have the added advantage of including the number of eligible voters (the electorate) of each PS, which is needed to calculate electoral turnout.

### Data access and final sample

To create a panel dataset of within-constituency voting patterns, we needed access to both the Form-20 data and ERs across states and over time. All of these documents are supposed to be publicly available. When our project started in 2013, we planned to collect data from all Indian states for the 2009 elections and then repeat the exercise for the upcoming 2014 elections.

We first located and downloaded all the documents available through the state-specific ECI websites but soon discovered that not all files were available for all states – either because they were not online at all or because there were problems with the links or with the posted documents. The hardest documents to access were the ERs for the 2009 elections: while these are public documents, they are regularly updated as the registered voters, catchment areas, and serial numbers of the polling stations change, and the huge number of files involved (one PDF per polling station means hundreds of thousands of PDFs) means that older versions are not necessarily kept.

**Fig. 2:**
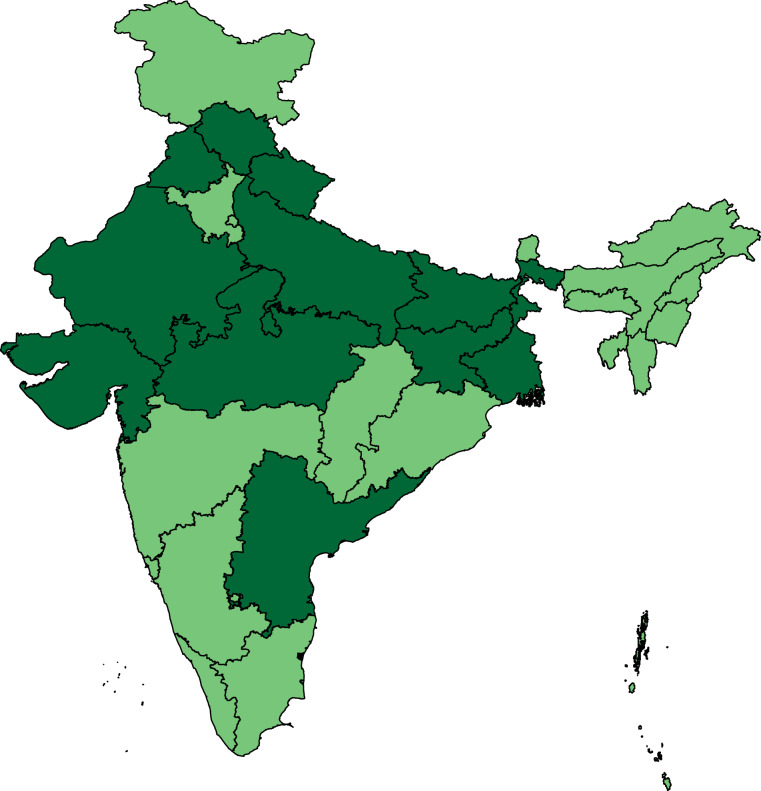
States included in the 2009 and 2014 data.

Having accessed all we were able to from active websites and internet archives, we contacted the relevant local ECI offices by mail, phone, or letter to access missing documents. After several rounds of contact, we were able to access Form-20 data and ERs for about 97% of the PSs in the 2009 elections for 10 states: Bihar, Gujarat, Himachal Pradesh, Jharkhand, Madhya Pradesh, Punjab, Rajasthan, Uttar Pradesh, Uttarakhand, and West Bengal (see Table 3). We also included Andhra Pradesh, for which we could access 95% of the PS data and administrative lists of the catchment areas of PSs, but not the ERs. This meant that we could match about 83% of the PSs in Andhra Pradesh to census units, but that we do not have the electorate for this particular state for the 2009 elections. The 11 included states cover about 57% of the Indian population. We were able to access both Form-20 data and ERs for most PSs in the same 11 states for the 2014 elections too. Figure 2 shows the 11 states that are included in the 2009 and 2014 data.

For the 2019 elections, we could also access most of the relevant files, but owing to the high cost of the data entry – which, as detailed below, had to be done manually to a large extent – we opted to focus on one state, Uttar Pradesh (UP) in Northern India. This state is the largest Indian state and is, according to the 2011 census, home to about 200 million people. It is also a crucial state for outcomes of Indian general elections because it elects approximately 14% (80 out of 543) of India’s parliamentarians. Uttar Pradesh is also covered extensively by the media during and after election campaigns, and it is the focus of a large share of academic studies of Indian politics. Since we had to choose just one state to look at, this therefore seemed a logical choice.

### Assembling the polling-station level data

Following acquisition of the Form-20 files, the data were digitized using R from all searchable PDFs or manually entered based on the non-searchable PDFs. Mostly, this parsing was unproblematic since the tables were formatted similarly across constituencies within states, so the code only needed minor adjustments across states.

**Fig. 3:**
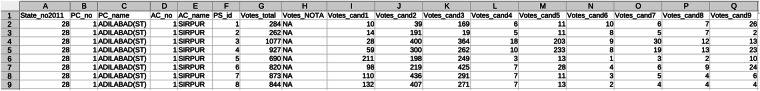
Excerpt from our version of the polling-station-level data for Andhra Pradesh from the 2009 elections.

To make it easier to merge data from different constituencies, states, and election years (which all have different candidates and candidate numbers), we removed candidate names and replaced them with a numeral (Candidate 1, Candidate 2 …). For polling stations within the same PC in the same election year, these columns refer to the same candidates, while across PCs and years, they differ. The data documentation includes candidate lists that facilitate merging the data with variables at the candidate level. In 2014 and 2019, the data include a column for the number of voters choosing “None of the above” (NOTA). This column is generally listed after the votes for each candidate in the Form-20 table. To allow that particular column to merge correctly across PCs with different numbers of candidates, we placed it before the column with votes for Candidate 1. We also included an empty column for NOTA votes in the 2009 data to facilitate data merging over time. Figure 3 shows how the data illustrated in Fig. 1 end up looking in our dataset.

### Linking polling stations to census indicators

In India, administrative units and political units do not have the same boundaries. Administratively, the country is divided into districts, sub-districts, villages (rural areas), and towns (urban areas). Each of these units has an identifier in the Indian census, and a large number of demographic and socio-economic indicators are collected at the level of these units. The political units in India are parliamentary constituencies, state assembly constituencies, and polling stations. One polling station sometimes overlaps perfectly with one village, but there are often several PSs in large villages or towns, while smaller villages generally share a single PS.

Whereas PS numbers change between the elections and no merge files exist to trace them over time, census units change less frequently, and the Indian census authorities release merge files to connect units across census rounds (most recently between the units in the 2011 census and the ones in the 2001 census), making it easier to trace units over time. Linking PS numbers to census indicators, therefore, has several advantages: it makes it possible to merge PS data with census data and to any other dataset that includes census indicators, and it also makes it possible to look at changes in electoral patterns at the census-unit level over time.

**Fig. 4:**
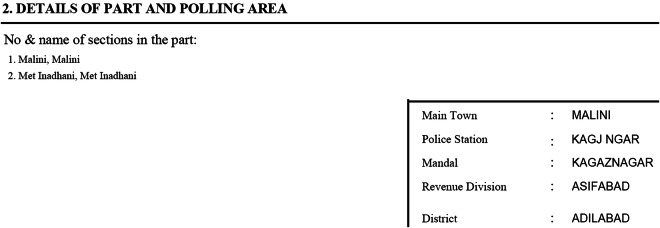
Example of the location information provided in an electoral roll from Adilabad district in Andhra Pradesh in 2014.

To add census identifiers to the PS-level data, we turned to the cover page of electoral rolls (ERs). There is one ER per polling station, and it lists every eligible voter. The cover page of each ER lists the total number of eligible voters in the PS (an important piece of information that is not included in the PS data) as well as information about the physical location of the PS: assembly constituency, sub-districts, and the village or town in which the PS is located. If multiple villages vote in the PS, this is also listed. As noted above, we were unable to access ERs for 2009 for Andhra Pradesh and instead relied on location lists for PSs for this particular state. Figure 4 provides an example of the location information provided on the cover page of the ERs. This example is PS 1 in Sirpur state assembly constituency in Adilabad district in Andhra Pradesh in the 2014 elections. The ERs from Andhra Pradesh for 2014 were the only ERs we worked with that were written in English the others were written in other Indian languages. The square on the right provides information about the village (main town), sub-districts (mandal), and district in which the PS was located, while on the top left we see a list of the villages that are included in the catchment area of the PS.

**Fig. 5:**
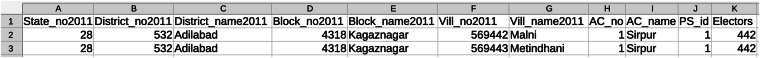
Example of the merge files generated on the basis of the location information provided in an electoral roll from Adilabad district in Andhra Pradesh in 2014.

On the basis of this information, we created merge files linking each polling station in our dataset to villages or towns in the 2011 census – the most recent census that has been conducted in India and also the census that was closest in time to our election data. For 2009 and 2014, this was done manually with the help of research assistants. Since most states publish ERs in local languages, we engaged a multilingual team of research assistants who could read the relevant parts of the ERs. Starting with a list of census units, research assistants would first identify the district and sub-district that a PS was in based on the names listed in the ERs, and then look for the names of villages and towns within the subset of villages and towns that are located within the relevant sub-district. If there were multiple PSs in a village or town, one row was created per PS so that there is at least one row in the merge file for each PS. When multiple villages vote in a PS, additional rows were added for each village. This means the merge files include duplicated rows for both PSs and villages and towns. Using this manual approach, we identified the location of 95% of the PSs in the 2009 data and 98% of the PSs in the 2014 data. Figure 5 shows how the data illustrated in Fig. 4 end up looking in our merge file.

Manually linking up polling stations to the census was both challenging and time-consuming. An alternative strategy would have been to overlay geographic information system (GIS) maps. For the 2009 elections, using GIS maps to link PSs and villages was not an option, as (to our knowledge) there are no geo-coded maps of where PSs were located in these elections. For 2014, maps do exist that provide the coordinates of polling stations, and it is possible to link them to the village and town they are located in by overlaying maps containing these points with polygons of villages and towns. However, since each PS then only overlaps with one village or town, there is no way of identifying the other villages that also vote in the same PS for all the villages that share a PS (in our 2014 data, that is 44% of villages in the data). Being able to map multiple villages perfectly to PSs is a clear advantage of the manual technique. Another advantage is that we do not have to rely on the accuracy of the geo-coded maps for villages and PSs, which have been found to have many errors. Raphael Susewind, who worked extensively with the maps in Uttar Pradesh found many irregularities^16^. A major political party in India, which also was using GIS maps to locate polling stations, professed to us that, in 2019, their accuracy rate was just over 50%.

Another strategy for linking PSs to census units is to use fuzzy name matching on the information provided in the ERs. This is challenging because of the various languages (with different scripts) used across Indian states and the fact that names are spelled differently across official documents. By 2019, however, the technology had advanced sufficiently for us to combine fuzzy name matching with our manual approach.

For the 2019 data, we first used the optical character recognition (OCR) package ‘tesseract’ for Python to read the names of the sub-district and village(s) or town(s) to which the PSs correspond from the first page of the ER for each polling station. The names were transliterated into Roman script using the Python package ‘indic_transliteration’. We then used fuzzy string matching (using the Python package ‘fuzzywuzzy’) to return the five villages from the corresponding sub-district in the census directory whose names most closely match the transliterated name from the ER, along with their village codes. We then manually selected the correct match from the five options returned. In other words, we did not use fuzzy name matching to select matches but rather to narrow down the list of potential matches from around 100 villages per sub-district to five for each identified village name, making the manual matching job much more cost-effective. Towns were matched manually since there is a limited number of towns in each district.

The main challenge when working with the 2019 data was that several census units had changed between 2011 and 2019 (particularly in urban areas), making it harder to match the names in the ERs to names in the census files. In the end we were able to identify matching villages and towns for about 86% of the PSs in the data.

## Data Records

Our data have been posted to Harvard Dataverse^17^. The PS-level election returns are saved by state and by year, using abbreviations for each included state: Andhra Pradesh (AP), Bihar (BH), Gujarat (GJ), Himachal Pradesh (HP), Jharkhand (JH), Madhya Pradesh (MP), Punjab (PJ), Rajasthan (RJ), Uttarakhand (UK), Uttar Pradesh (UP), and West Bengal (WB). The different files can easily be merged since they are all structured the same way. Table 1 includes a codebook for the included variables. The merge files are also saved by state and by year. Table 2 includes a codebook for the variables in the merge files.

**Table 1:** Codebook for the polling-station-level election returns.

Variable name	Variable description
State_no2011	The state number as provided by the Indian Census of 2011.
PC_no	The number of the parliamentary constituency that the polling station is part of, as given by the Election Commission of India. This number is unique within each state and can be merged with other election data.
PC_name	The name of the parliamentary constituency that the polling station is part of.
AC_no	The number of the state assembly constituency that the polling station is part of, as given by the Election Commission of India. This number is unique within each state and can be merged with other election data.
AC_name	The name of the state assembly constituency that the polling station station is part of.
PS_id	The polling station number (this may change between elections). These numbers are mostly integers, but in a few cases where polling stations have been split into two or more booths, the integers are followed by a letter. Remove the letter and add the votes for two parts of a polling station to merge with the merge files.
Votes_total	The total number of valid votes cast in this polling station.
Votes_NOTA	The number of votes cast for “None of the above” in this polling station (this variable is NA for 2009 since there was no NOTA voting in these elections).
Votes_cand1	The number of votes cast for Candidate number 1 in the parliamentary constituency that the polling station is part of. Note that the candidates are usually ordered in the way they appeared on the ballot (not by final vote share). The Form-20 data can be consulted if in doubt.
...	The number of votes cast for Candidate number ... in the parliamentary constituency that the polling station is part of.
Votes_cand43	The number of votes cast for Candidate number 43 in the parliamentary constituency that the polling station is part of.

**Table 2:** Codebook for the merge files linking polling stations with census identifiers.

Variable name	Variable description
State_no2011	The state number as provided by the Indian Census of 2011.
District_no2011	The district number as provided by the Indian Census of 2011.
District_name2011	The district name as provided by the Indian Census of 2011.
Block_no2011	The block (sub-district) number as provided by the Indian Census of 2011.
Block_name2011	The block (sub-district) name as provided by the Indian Census of 2011.
Vill_no2011	The 2011 census number of the village or town the polling station is in (coded on the basis of information in the electoral rolls).
Vill_name2011	The 2011 census number of the village or town name the polling station is in (coded on the basis of information in the electoral rolls).
AC_no	The number of the state assembly constituency that the polling station is part of, as given by the Election Commission of India. This number is unique within each state and can be used to merge with other election data.
AC_name	The name of the state assembly constituency that the polling station is part of.
PS_id	The polling station number (may change between elections). Together with the AC_no, this can be used to merge these files with the polling-station-level data (using AC_no and PS_id).
Electors	The number of registered voters in a polling station (taken from the electoral rolls).

Our dataset includes election returns from 495,334 PSs from 2009. This includes most of the PSs in the 11 included states (and all the ones we were able to access data from). Table 3 shows the official number of polling stations for each state in the dataset, the number of PSs in our data, the average number of votes cast in these PSs, the percentage of PSs in our data that are linked to census identifiers, and the number of villages and towns included when the data are collapsed to the census-unit level. In a few cases (Punjab and West Bengal), our data include more PSs than the official number of PSs in the state. This is probably because some PSs split after the official numbers had been recorded.

**Table 3:** Summary statistics for the 2009 data.

State name	Number of polling stations	Number of PSs in data	Average number of votes cast	% PSs linked to census	Number of census units
Uttarakhand	9300	8991	347	99.5	15210
Jharkhand	23696	23509	381	94.8	26352
Rajasthan	42699	42478	420	99.7	42034
Punjab	18846	18850	628	98.3	10196
Uttar Pradesh	129446	122939	429	94.7	64535
Madhya Pradesh	47812	43277	411	99.6	48402
Bihar	57020	56224	423	95.9	35002
Himachal Pradesh	7253	7251	371	99.9	18874
Gujarat	42568	42046	408	95.6	16350
Andhra Pradesh	66760	63646	629	82.6	19612
West Bengal	66109	66123	646	99.3	34225
**Total**	**511509**	**495334**	**482**	**95.1**	**330792**

*Note*: The figures for the number of polling stations by state are from page 123 of the *General Elections 2024* by the Indian Ministry of Information and Broadcasting^18^.

As there were 830,866 PSs across India in 2009^18^, the data included in our 2009 dataset amounts to 60% of the PSs in India. In these data, the average number of votes cast in a PS is 482. We were able to link 95% of the included PSs to census indicators. When collapsed to the census-unit level, the data include 330,792 villages and towns. Out of the villages in the data, 41% shared a polling station with at least one other village, about 37% had one polling station, and the remaining 22% of the census units – larger villages and towns – had more than one polling station. The largest cities have the highest number of polling stations, and some cities even span several parliamentary constituencies.

For 2014, our dataset covers 528,580 PSs. This was again all the PSs for which we were able to access data from our 11 included states, and it amounts to about 57% of the 927,553 PSs in India that election year^18^. Here, the average number of voters in a polling station is 588. We identified the corresponding villages and towns for 98% of these polling stations and there are on average 1.5 polling stations to a census unit. When collapsed to the census-unit level, the dataset covers 358,759 villages and towns. In these data, some 45% of the villages shared a polling station with at least one other village, about 33% had one polling station, and the remaining 22% of the census units had more than one polling station. Table 4 shows the official number of PSs in each state, the number of PSs in our data, the average number of voters across these PSs, the percentage of PSs linked to a census identifier, and the number of villages and towns included in the census-unit-level version of the data.

**Table 4:** Summary statistics for the 2014 data.

State name	Number of polling stations	Number of PSs in data	Average number of votes cast	% PSs linked to census	Number of census units
Uttarakhand	10078	10103	433	98.6	15224
Jharkhand	24751	24745	525	99.4	29683
Rajasthan	47947	47442	562	99.1	39774
Punjab	22019	22024	628	96.8	11694
Uttar Pradesh	140485	140259	577	100	99766
Madhya Pradesh	54844	53129	537	99.8	52564
Bihar	61721	60796	581	91.6	32701
Himachal Pradesh	7385	7105	423	99.7	18227
Gujarat	45383	45386	566	99.6	18030
Andhra Pradesh	71225	40922	684	98.9	16433
West Bengal	77252	76669	667	97.6	24663
**Total**	**563090**	**528580**	**587**	**98.3**	**358759**

*Note*: The figures for the number of polling stations by state are from page 123 of the *General Elections 2024* by the Indian Ministry of Information and Broadcasting^18^.

For 2019, our dataset includes election returns from 161,843 polling stations in Uttar Pradesh. This is 99.5% of the polling stations in Uttar Pradesh in those elections (about 16% of the polling stations in India). We were able to identify the corresponding census units to 86% of these PSs. On average, 528 votes were cast in each PS and there were about 1.7 polling stations to a census unit: about 32% of the villages had exactly one corresponding polling station, 34% census units had more than one polling station. When collapsed to the census-unit level, the dataset includes 83,031 villages and towns.

## Technical Validation

We ran various quality assurance checks on the PS-level data and the merge files to make the files as accurate as possible. For the PS-level data, we checked whether any numbers were missing between 1 and the largest serial number for polling stations in the constituency and whether the votes that each candidate received across PSs aggregated correctly to the number of votes they got in a constituency. Where this was not the case, it was generally because some PS data were missing, either because a whole PDF that should have included data was missing or empty or because a particular PS was missing from the data provided. In these cases, we made efforts to add the missing PSs, though this was generally not possible. As shown in Table 3, we were able to include data on about 97% of the PSs in the states in the 2009 sample. In 2014, our PS data include about 94% of the PSs in the states (most of the missing data were in Andhra Pradesh). For UP in 2019, our PS-level data include 99.5% of the PSs in the state in those elections.

**Fig. 6:**
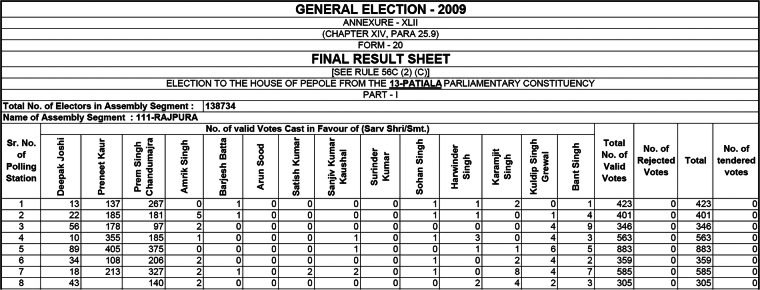
Example of missing data in the original data files (number of votes for candidate 2 in polling station 8).

We also ran a row-by-row check of all the PS data to check whether the votes for each candidate in the PS added up to the total number of votes in the PS and that there were no negative values, letters, or punctuation in the columns listing numbers of votes. Any data row that was marked as potentially problematic in these automated tests was manually checked against the original Form-20 data and corrected when needed. In the rows where the numbers do not add up correctly, this is generally because the numbers do not add up correctly in the original files, because the quality of PDFs that are scans is too poor for us to be certain about all the numbers, because or because numbers are missing. We have provided an example of the latter issue in Fig.6.

For the merge files, we double-checked all matches where the villages and towns of consecutive polling stations were in different sub-districts or far from each other geographically. We also double-checked the matches where census villages appeared in more than one state assembly constituency (they are normally only in one), and where there were large discrepancies between the population of a census unit and the eligible voters in the PSs matched to it. Usually, there was no mistake in the matches that had been identified (it is, for example, often the case that consecutive polling stations are located in different sub-districts), but where we did identify a problem, the matches were corrected. All the checks were conducted manually by the research assistants or by one of the authors.

## Usage Notes

The data can be used at the polling-station level or merged with the census identifiers to be used at the census-unit level. At the polling-station level, data from different states can be added simply using the command ‘rbind’ in R (the R code is included in the dataset’s documentation).

Each PS-level file can be merged with the corresponding census merge file using the identifiers for state assembly constituencies (AC_no) and polling stations (PS_id). Note that villages and towns in the merge files may correspond to multiple PSs (and several villages may be matched to one polling station). Therefore, the data need to be collapsed to an appropriate level of analysis after being merged. R code collapsing the data to the census-unit-level included in the documentation of the dataset^17^.

Collapsing the data to the census-unit level makes it possible to examine changes in voting patterns for the same units over time. The census identifiers in the merge files can be used to merge in any dataset that includes identifiers from the 2011 Indian Census based on the identifiers for states, districts, sub-districts, and village (or towns). By using the indicators for state assembly constituency and parliamentary constituency, the data can also be merged with any dataset that includes constituency identifiers for the post-2008-delimitation period.

The vote shares for different candidates across PSs (or census units) can be used to create variables such as the local-level vote fragmentation but not to compare vote shares by candidate or party across PCs since the candidates running for election in different PCs differ. For such comparisons, the data must be merged with constituency-level candidate information. In the dataset documentation^17^, we have included candidate lists that facilitate the merge with candidate-level data. For 2014 and 2019, the *Cand*_*no* variable corresponds to the number the candidate has in the PS data, and *Cand*_*position* is the position they got in the elections. Note that since we have moved NOTA votes to appear before the votes for other candidates in all our datafiles, NOTA has not been included in these lists and must be merged separately. For 2009, most of the Form-20 data were ordered by candidate number, but for some ACs in Uttar Pradesh the PS-data were reported by the position of the candidates in the elections. For this reason, we have included a separate candidate list for UP, which includes AC indicators and also the extra variable *Ordered*_*by*_*position* which is 1 if the candidates in that AC were ordered by *Cand*_*position* and 0 if they were ordered by *Cand*_*no*. This indicator can be used to merge candidates for all the PSs in the relevant ACs correctly to PC level candidates using either *Cand*_*no* or *Cand*_*position*.

## Data Availability

Included in our data release are three R codes (for 2009, 2014, and 2019)^17^, each merging all the PS-level files for their respective years together, merging all merge files together, merging the PS-level files with the merge files, and collapsing the files to the level of census units. The summary statistics reported in this data descriptor can be reproduced using these codes. In the folder with supplementary documents, we also include the script for extracting information from the cover page of the 2019 electoral rolls, for the transliteration of village names into English, and for the fuzzy matching.
